# Inductive and Deductive Methods of Study

**Published:** 1879-11

**Authors:** E. G. Betty

**Affiliations:** Cincinnati


### INDUCTIVE AND DEDUCTIVE METHODS OF STUDY.

BY E. G. BETTY, D. D. S., CINCINNATI.

In every field of inquiry there is always found one or more thinkers, who far ahead of the general intelligence of their age, advance doctrines that appear to be insane to their contemporaries.

This was more frequently the case during the last few hundred years than in the present century, though this age has also its scientific speculators.

Great difference of opinion has always existed, even between men of vast intellect and acknowledged ability, and the confusion arising from opposing ideas, is sufficient to bewilder any one not given to continued study. Though all philosophers agree that truth is the great object of their endeavors, yet it is often difficult, and frequently impossible to reconcile the conclusions of one with the deductions of another, notwithstanding the fact that, the one and same truth is the avowed desideratum of both.

The difficulty seems to originate in the method by which the truth is sought. The inductive method, of which Aristotle, was the great expounder, beginning with detailed facts and experiences, acquired only after long and careful research into the properties of matter and effects of force, passes by a gradual process from details to generalities.

The deductive method, on the contrary, presupposes the existence of fundamental principles, all subsequent investigation and experiment, only seeking to adapt the fact or facts to the initial principle. This latter method, is to many, faulty. First, because principles can only be acknowledged and established as such, by previous experience. Secondly, the endeavor to adapt facts to the demands of a theory, gives evidence that the method is radically erroneous. The inductive method, is that which in the present age appears to be most generally accepted, a strict adherence to it, having caused many of the greatest intellects of our time to be denounced as materialists. Be that as it may, it can not in any way detract from the merits and value of a system which has placed man in a position that enables him to modify and adapt his surroundings to his own convenience and advancement.

According to Buckle, we can not arrive at absolute truth, until the two methods have been so perfected that they will harmonise, and uniformly be the means of securing the same results. To show that one result is sometimes reached by both methods, he instances the discovery of the composition of water almost simultaneously, by Jas. Watt, of Scotland, and Cavendish, of England.* Watt's statement of the case was by deduction, and he was firm in his belief, though he had not at the time, tried to prove it by demonstration.

*History of Civilization in England, Vol., II., pp. 414, 415, 416.

That was reserved for the English chemist, who having no intimation of Watt's idea, discovered the fact after a series of laborious investigations.

A good illustration of the discord, generally arising from these conflicting methods, may be seen in our attempts to demonstrate the causes producing dental caries.

Without going into details which are well known to all intelligent dentists, it may be stated that the circumstances inducing caries, are classed under two heads, predisposing and acting. It is the purpose here to notice the methods by which the latter are accounted for, and if possible to show wherein the recently advocated electrical theory, clashes with all previously established facts.

For a series of years back, investigations set afoot for the purpose of throwing light upon the causes of caries, have resulted in the accumulation of a vast number of detailed facts, the classification of which has enabled us to get some idea of laws operating to produce them. Each variety of decay was thus found to be governed by its own law, and from a diligent comparison of these minor laws, there has finally arisen the grand conception of the all-pervading control of chemical affinity.

To Dr. Geo. Watt, (who by the way, is a grand nephew of the great Scotch physicist,) is due the credit of having been the first to promulgate this doctrine in a concise and perspicuous manner. The young man of to day, who half a century hence, when time has bent his form and silvered his hair, will be proud to say that, in his youth he looked upon the kindly face of Dr. Watt, and heard from his own lips, an exposition of a grand and exalted truth. It will thus be seen that the chemical theory has its foundation in facts, and from them the conclusion was reached by a process of induction, slow, but sure. In opposition to this accepted doctrine, a new theory has been advanced within the last two or three years, to the effect that electrical action is the cause or force producing decay of the teeth. Taking this for their principle, the advocates of the new heresy, are making strenuous efforts to compel facts to substantiate it.

If the theory be true then the process must necessarily be by electrolysis, otherwise electricity can have nothing to do with the production of dental caries. The text-books teach that fluidity is absolutely essential for decomposition by the electric current and it is certainly beyond comprehension that a tooth which is a solid body, can by any means become an electrolyte.

Every student knows that when two metals united by a conductor are immersed in a fluid which acts upon them unequally, chemical force immediately ceases as such, the evolution of electricity resulting. Such conditions are frequently found in the mouth, a gold filling actually in contact or in close proximity with another of tin or amalgam.

In this case whatever chemical action may take place when a solvent of one of the metals is presented or formed, immediately becomes electrical.

As the decaying agent, *i. e.* acid, is always formed in the mouth from the products of decomposition, we can readily see that chemical action is the force which produces, first, the destruction of foreign bodies lodged between the teeth; second, the immediate formation of new compounds; third, the action of these compounds upon the teeth. If during these processes too metals be introduced into the reacting fluids the circumstances are necessarily changed and the conversion of chemical force into electrical instantly takes place.

It is highly probable that the presence of electricity thus originated has been mistaken by some as the cause operating to produce decay; if not, why are we confronted with tables showing the degrees of electrical tension manifested when this, that or the other filling material is introduced into the mouth? It is not denied by any one that galvanism may occur in the oral cavity, but the proof is lacking to show that it is the initial force. The torpedo fish possesses a regularly constituted electric battery, but as we have yet to learn that one also exists within the animal economy of the *genus homo*, the advocates of the new departure, electrical theory etc., must still be content to depend upon chemical action for the formation of their galvanic current.

It is useless and puerile to charge the new departurists with dishonesty, for they are conscientious in their belief and are really trying to advance our knowledge upon a subject that is of the highest importance and interest to all of us.

It is not wise to deride a man for opinion's sake; we forget that we do not see things as he does and were it not for the different ideas we individually entertain and the methods by which we pursue our studies and investigations, civilization would cease to progress and intellectual development be a thing unknown.

